# Antioxidant and Cytotoxic Potential of *Carlina vulgaris* Extract and Bioactivity-Guided Isolation of Cytotoxic Components

**DOI:** 10.3390/antiox12091704

**Published:** 2023-09-01

**Authors:** Ireneusz Sowa, Roman Paduch, Jarosław Mołdoch, Dariusz Szczepanek, Jacek Szkutnik, Paweł Sowa, Katarzyna Tyszczuk-Rotko, Tomasz Blicharski, Magdalena Wójciak

**Affiliations:** 1 Department of Analytical Chemistry, Medical University of Lublin, Chodźki 4a, 20-093 Lublin, Poland; 2 Department of Virology and Immunology, Institute of Biological Sciences, Faculty of Biology and Biotechnology, Maria Curie-Skłodowska University, 19 Akademicka Street, 20-033 Lublin, Poland; roman.paduch@mail.umcs.pl; 3 Department of Biochemistry and Crop Quality, Institute of Soil Science and Plant Cultivation, State Research Institute, 24-100 Puławy, Poland; jmoldoch@iung.pulawy.pl; 4 Chair and Department of Neurosurgery and Paediatric Neurosurgery, Medical University of Lublin, 20-090 Lublin, Poland; dariusz.szczepanek@umlub.pl; 5 Independent Unit of Functional Masticatory Disorders, Medical University of Lublin, 20-093 Lublin, Poland; jacek.szkutnik@umlub.pl; 6 Department of Otorhinolaryngology and Oncological Laryngology, Faculty of Medical Sciences in Zabrze, Medical University of Silesia in Katowice, 40-055 Katowice, Poland; psowa@sum.edu.pl; 7 Institute of Chemical Sciences, Faculty of Chemistry, Maria Curie-Skłodowska University, 20-031 Lublin, Poland; katarzyna.tyszczuk-rotko@mail.umcs.pl; 8 Department of Orthopaedics and Rehabilitation, Medical University of Lublin, 20-954 Lublin, Poland; blicharski@vp.pl

**Keywords:** carline thistle, human colorectal adenocarcinoma, cancer, oxylipins, traumatic acid, pinellic acid

## Abstract

*Carlina vulgaris* is a poorly understood plant in the context of biological activity, despite its widespread application in ethnomedicine in numerous European countries. The aim of this study was to assess the cytotoxic potential of the plant against human colorectal adenocarcinoma (HT29) and to isolate the plant components linked to this effect. Ultra-high performance liquid chromatography with a high-resolution/quadrupole time-of-flight mass spectrometer (UHPLC–HR/QTOF/MS–PDA) was used for the phytochemical characterization of the extract. Liquid–liquid extraction and preparative chromatography were employed for fractionation purposes. Our investigation demonstrated that the ethyl acetate fraction from *C. vulgaris* showed significant cytotoxicity, and a bioactivity-guided approach led to the isolation of oxylipins, including traumatic acid, pinellic acid, and 9,10-dihydroxy-8-oxsooctadec-12-enic acid. The structures of the compounds were confirmed by nuclear magnetic resonance spectroscopy. Among these compounds, the last one exhibited significant cytotoxicity, though without selectivity, and traumatic acid was characterized by mild cytotoxicity. The cytotoxicity was linked to intracellular reactive oxygen species generation.

## 1. Introduction

The plant kingdom represents a vast reservoir of biologically active molecules; therefore, plants and products of natural origin have been used in traditional medicine since ancient times [1]. Currently, there is a wealth of scientific evidence confirming their traditional usage as effective agents in the treatment of various diseases [2]. However, despite significant progress in this field, the potential of many species still remains unexplored. Among these species, plants from the *Carlina* genus belong to those poorly understood in the context of biological activity, despite their widespread application in the ethnomedicine of numerous European countries, mostly in gastrointestinal dysfunctions and externally to treat skin disorders [3,4]. Recently, *C. acaulis* has aroused the interest of scientists because of carlina oxide, the main volatile component of its essential oil, which has shown a strong insecticidal effect and, thus, has potential as a natural pesticide [5,6]. Furthermore, evidence has shown that the species exhibits antioxidant, antibacterial, antifungal, and anti-ulcer properties [7,8,9]. Additionally, it has been found to be cytotoxic against a few types of cancer cell lines [10,11,12].

In turn, *C. vulgaris* L. is a less studied species from the *Carlina* genus. Phytochemical studies have shown that *C. vulgaris* contains pentacyclic triterpenes, chlorogenic acid, and flavonoid C-glycosides [13,14,15]. The essential oils are rich in polyacetylenes, with the most abundant being carlina oxide (33.7%) and 13-methoxy carlina oxide (11.5%) [16]. However, knowledge regarding the biological activity of *C. vulgaris* is scarce. To date, it has been found that the plant extracts display a free radical scavenging effect in ABTS and DPPH tests. Our previous study also revealed that they possess strong antioxidant activity and a protective effect against H_2_O_2_-induced oxidative stress in human skin fibroblasts [15]. Furthermore, the essential oil showed antioxidant and antifungal activity against *Penicillium expansum* and *Aspergillus niger* [16].

Here, we investigated the potential of the plant and plant components against human colorectal adenocarcinoma (HT29), based on a bioactivity-guided approach.

## 2. Materials and Methods

### 2.1. Reagents and Standards

Phenolic compound analytical standards and chemicals like 2,2-diphenyl-1-picrylhydrazyl (DPPH), o-phenanthroline, and ferric chloride (FRAP reagent) were acquired from Fluka (Sigma-Aldrich Co., St. Louis, MO, USA). LC–MS grade methanol, acetonitrile, and formic acid were procured from Merck KGaA (Darmstadt, Germany). Other solvents used were of analytical grade from Merck. Water was deionized and purified through an Ultrapure Millipore Direct-Q^®^ 3UV-R system (Merck KGaA, Darmstadt, Germany).

### 2.2. Plant Material

Plants of *Carlina vulgaris* were obtained from the UMCS Botanical Garden in Lublin (voucher specimen no. 9/2009S). The collection took place in August 2019, precisely during the flowering phase. After collection, the plant material underwent a series of steps, including washing with running water, subsequent drying, freeze drying, and stored at a temperature of −20 °C until further processing.

### 2.3. Extraction, Fractionation, and Subfraction

The above-ground parts of the plants were ground, and 300 g of material was extracted sequentially with methanol and 70% methanol (3 × 5 L and 3 × 1.5 L for 15 min each), using an ultrasonic bath. The extracts (ECV) were combined, centrifuged at 8000 RPM, and filtered and then, concentrated using a vacuum evaporator, frozen, and subjected to freeze drying. The freeze-dried ECV was suspended in 300 mL of MeOH and subjected to liquid–liquid extractions with *n*-hexane (5 × 100 mL) (HCV fraction), followed by ethyl acetate (5 × 100 mL) (EaCV fraction). The residue was evaporated to dryness and suspended in water, then extracted with *n*-butanol (5 × 100 mL) (BCV fraction). The residue after extraction represented the H_2_OCV fraction. The fractions were concentrated using a vacuum evaporator, frozen, and freeze dried. After conducting the bioactivity test, the most potent fraction (EaCV) underwent further separation. The fraction was mixed with deionized water and 5% DMSO, followed by centrifugation (1000× *g*; 10 min). The resulting liquid was placed onto a glass preparative column (10 × 30 cm) filled with Cosmosil 75C18-PREP chromatographic material (75 μm, Nacalai Tesque, Kyoto, Japan) that had been pre-impregnated with 1% MeOH in water. Around 10 g of the dissolved extract was loaded onto the column at once. Elution was carried out using a stepwise gradient of the mobile phase, with each concentration equivalent to 3 times the column volume. Elution involved using 20%, 60%, and 100% MeOH to obtain distinct fractions: EaCV_1 (20% MeOH), EaCV_2 (60% MeOH), and EaCV_3 (100% MeOH). The extraction procedure is presented in the Supplementary Materials, Scheme S1.

### 2.4. Chromatographic Analysis (UHPLC–HR/QTOF/MS–PDA)

The samples were subjected to qualitative chromatographic analysis using an ultra-high performance liquid chromatography (UHPLC) system (Dionex UltiMate 3000RS, Thermo Scientific, Waltham, MA, USA), coupled with a high-resolution/quadrupole time-of-flight mass spectrometer HR/QTOF/MS Impact II (Bruker, Billerica, MA, USA), employing electrospray ionization (ESI). The chromatographic separation was carried out using a BEH C18 column (2.1 × 150 mm, 1.7 µm; Waters, Milford, MA, USA) at 40 °C. A gradient elution with a linear profile was employed, maintaining a consistent flow rate of 0.5 mL/min, using solvent B (acetonitrile-0.1% formic acid (FA)) in solvent A (H_2_O-0.1% FA), ranging from 2% B to 80% B over 30 min. The UV spectra of the compounds were recorded from 190 to 750 nm, with a resolution of 3.6 nm. The MS spectra were acquired using the negative ion mode, scanning across the range of 100–1200 *m*/*z*. Nitrogen gas was used as the cone and spraying gas, with flow rates of 800 L/h and 100 L/h, respectively. The capillary voltage was set at −2.8 kV for the negative mode. The cone voltage was −25 V and 45 V, respectively, the source temperature was 140 °C, and the spraying gas temperature was 350 °C. The acquisition and processing of the data were carried out utilizing Waters MassLynx software ver. 4.1. Quantitative analyses were executed employing an ultra-performance liquid chromatography system UPLC-PDA-ESI-MS (ACQUITY, Waters), which was coupled with a PDA detector and a triple quadrupole mass spectrometer (ACQUITY TQD, Waters). The chromatographic conditions were mentioned above.

### 2.5. Isolation of the Active Compounds

The isolation of the active compounds was performed using a semi-preparative HPLC system GX-271 (Gilson Inc. Middleton, WI, USA) equipped with a PrepELS™ II evaporative light scattering detector (Gilson Inc.) on an Atlantis^®^ T3 Prep OBD™ column (19 × 250 mm, 5 μm, Waters), at a temperature of 50 °C with a flow rate of 12 mL/min. The eluent used was 28% ACN in water with the addition of 0.1% FA. The injection volume was 1.8 mL, and the fraction concentration was 80 mg/mL. The total duration of isocratic elution was 110 min. The PrepELS™ II detector had the following settings: a drift tube temperature of 65 °C and the nebulizing gas was nitrogen supplied at a pressure of 47 psi. The eluent from the HPLC system was split before the detector at a ratio of 1:100. The eluate was automatically collected using the GX-271 liquid handler fraction collector (Gilson Inc.), with each fraction collecting 12 mL. The fractions were checked using the UHPLC method described in Section 2.4. Fractions containing the same compounds were combined, the organic solvent was evaporated under reduced pressure, followed by freeze drying, and then further investigated for their activity.

### 2.6. Nuclear Magnetic Resonance (NMR) Spectroscopy

The results of the spectral analyses from the high-resolution mass spectrometry (UHPLC-ESI-MS/MS), as well as the one-dimensional nuclear magnetic resonance (1D NMR), were utilized for the assessment of the chemical structure of the isolated compounds. One-dimensional NMR experiments were conducted using a Bruker Avance II HD Ascend™ 500 spectrometer (^1^H, 500.20 MHz; ^13^C, 125.80 MHz; Bruker), equipped with a 5 mm broadband inverse probe for 1H. Deuterated methanol (MeOH-d4) was used as the solvent. The NMR experiments were carried out at a temperature of 30 °C, employing standard parameters and pulse sequences for recording the 1D spectra (^1^H, ^13^C). Chemical shifts (δ) were reported in ppm units, and coupling constants (J) were in Hz, referenced to residual methanol (δH 3.31 and δC 39.5). Topspin software (version 3.5pl2, Bruker) was employed for the data analysis.

### 2.7. Antioxidant Activity

#### 2.7.1. DPPH Radical Scavenging Assay

The 2,2-diphenyl-1-picrylhydrazyl (DPPH) assay was carried out according to the procedure published previously [15]. Fraction/subfraction/compounds were dissolved in methanol, diluted, and mixed with a 4 mM methanolic DPPH solution. The absorbance was measured at the wavelength λ = 517 nm, using a UV–VIS Filter Max 5 spectrophotometer (Thermo Fisher Scientific, Waltham, MA, USA). Water with a DPPH solution was used as a control. The calibration was based on Trolox as the standard.

#### 2.7.2. Ferric Ion Reducing Antioxidant Power (FRAP Assay)

The ferric reducing activities of the samples were determined according to the method described by Sowa et al. [17] with some modifications. Fraction/subfraction/compounds were dissolved in methanol and diluted with water. The sample (15 μL) was mixed with fresh FRAP reagent (350 μL). The 300 mM acetate buffer pH 3.6, 10 mM TPTZ in 40 mM HCl, and 20 mM FeCl_3_·6H_2_O were mixed according to 10:1:1 (as the FRAP reagent), and reacted in the dark for 5 min. The absorbance was measured at λ = 593 nm. The calibration was based on ascorbic acid as the standard.

### 2.8. Cell Culture

The human colorectal adenocarcinoma (HT29) cell line (ATCC^®^ No. HTB-38™) was applied in this research. The cells were cultured in RPMI 1640 medium with 10% fetal calf serum (FCS) (GibcoTM, Paisley, UK) and antibiotics (100 U/mL penicillin, 100 μg/mL streptomycin, and 0.25 μg/mL amphotericin B) (GibcoTM, Paisley, UK), at 37 °C in a humidified atmosphere with a flow of 5% CO_2_. The normal human colon epithelial cell line (CCD 841 CoTr) (ATCC^®^ No. CRL-1807™) was used as a reference line. These cells were cultured in RPMI 1640 + DMEM (1:1) medium mixture (Sigma-Aldrich Co. LLC, St. Louis, MO, USA), with the addition of 10% FCS and antibiotics at 37 °C in a humidified atmosphere with a flow of 5% CO_2_. As the cell viability assay was performed, the cells (1 × 10^5^ cells/mL) were plated in 96-well flat-bottom plates, incubated for 24 h at 37 °C, and then treated with the tested compounds for 24 h. A stock solution of the tested substances was prepared using a mixture of the DMSO/culture medium (1:1) and appropriately diluted. The final concentration of the DMSO did not exceed 0.5% at the highest applied working concentration. Cells treated only with 0.5% of the DMSO in the culture medium were considered as the control. All the experiments were performed in triplicate for each extract concentration and presented as a percentage of the control (predetermined as 100%).

#### 2.8.1. MTT Assay

After 24 h of incubation with the tested compounds, a 3-(4,5-dimethylthiazole-2-yl)-2,5-diphenyltetrazolium bromide solution (MTT) was added 5 mg/mL (Sigma) to the cells (25 μL/well). Thereafter, 3 h of incubation at 37 °C was performed. After that time, the 10% sodium dodecyl sulfate (SDS) in 0.01 M HCl solution to dissolve formazan crystals. Solubilization was allowed to proceed overnight. Absorbance was measured at the 570 nm wavelength using an E-max Microplate Reader (Molecular Devices Corporation, Menlo Park, CA, USA).

#### 2.8.2. Neutral Red Uptake Assay

After 24 h of incubation with the tested compounds, the medium was removed from the wells, and a solution with a neutral red dye (NR) (40 μg/mL) (100 μL/well) was added. A further 2 h of incubation at 37 °C was continued. After this time, the NR solution was removed and the cells were washed with phosphate buffered saline (PBS). The PBS was removed, the cells were fixed with 200 µL of 0.5% formalin in 1% CaCl_2_ and, then, 150 µL of decolorizing buffer (1% glacial acetic acid in 50% ethanol) was added. The plates were shaken for 10 min, and the optical density (OD) of the eluted dye was measured at 540 nm using an E-max Microplate Reader (Molecular Devices Corporation, Menlo Park, CA, USA). The results are presented as the percentage of the amount of dye retained compared to the control cells (predetermined as 100%)

#### 2.8.3. Detection of Intracellular Levels of Reactive Oxygen Species (ROS)

The fluorogenic H_2_DCFDA probe was used to assess the intracellular levels of reactive oxygen species [18]. H_2_DCFDA was added to the cells and incubation in the dark for 45 min was performed. Thereafter, the tested compounds at concentrations of 100 and 200 μg/mL were added. The cells treated with 500 μM hydrogen peroxide (H_2_O_2_) were taken as a positive control. After 60 min of incubation, the fluorescence of 2′,7′-dichlorofluorescein (DCF) was measured. The measurements were performed at an excitation wavelength of λ = 485 nm and an emission wavelength of λ = 530 nm, using a microplate reader (FilterMax F5, Thermo Fisher Scientific, Waltham, MA, USA). Three independent experiments were performed.

#### 2.8.4. Cellular Morphology Analysis, May–Grünwald–Giemsa (MGG) Staining

This method was used to illustrate the changes that occurred in the cell morphology under the influence of the tested compounds. The staining was performed on 24-well plates. The cells at a density of 1 × 10^5^ cells/mL were used. After 24 h of incubation with the examined extracts or without them (control), the culture media was removed and the cells were stained with 1 mL of May–Grünwald dye for 3 min at room temperature. Then, 1 mL of deionized water was added to each plate. After 3 min of incubation at room temperature, the liquid was removed. All the plates were rinsed with deionized water, and then the cells were stained with Giemsa dye (dilution 1:20) for 30 min at room temperature. After this time, the dye was removed and the wells were rinsed with 1 mL of deionized water and then allowed to dry. The images were taken using an Olympus BX51 light microscope.

### 2.9. Statistical Analysis

All analyses were replicated three times. The results were subjected to analysis utilizing Statistica ver. 13.3 software. A one-way ANOVA was conducted, followed by Dunnett’s post hoc test. The data were presented as the mean ± standard deviation (SD). The differences were considered significant at a *p*-value of < 0.05.

## 3. Results

### 3.1. Cytotoxicity Assessment of C. vulgaris Fractions

**Figure 1:**
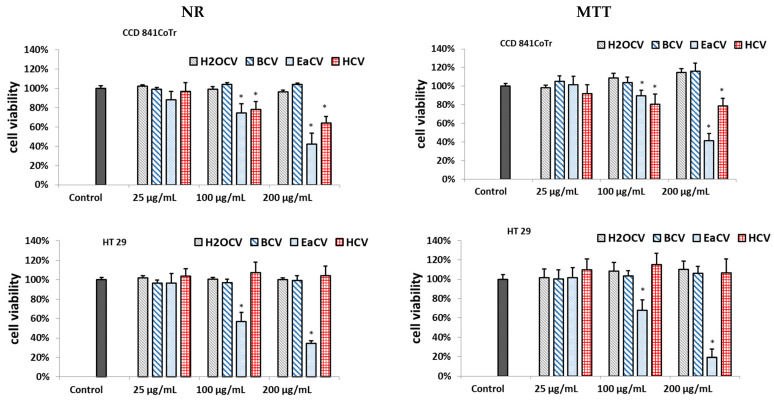
Effect of the different concentrations of fractions obtained from methanol/water extract of *Carlina vulgaris* on cell viability determined by the NR and MTT assays and expressed as a % of the control (0.5% of DMSO in medium). The fractions were obtained using hexane (HCV), followed by ethyl acetate (EaCV), butanol (BCV), and water (H_2_OCV). The data are the mean (*n* = 3) ± SD. One-way ANOVA followed by Dunnett’s post hoc test; the differences between the samples and the control were considered significant at *p* < 0.05. * Indicates statistically significant difference.

The cytotoxicity of the methanol water extract (ECV) and the *n*-hexane (HCV), ethyl acetate (EaCV), *n*-butanol (BCV), and water (H_2_OCV) fractions against human colorectal adenocarcinoma (HT29) was investigated using two complementary tests: neutral red (NR) and MTT. Normal human colon epithelial cells (CCD 841 CoTr) were used as a reference line. The results are presented in Figure 1 and Figure S1.

Among the fractions, EaCV demonstrated significant cytotoxicity; however, it affected the viability of both the normal and colorectal adenocarcinoma cells. Nevertheless, it had a significantly greater impact on the HT29 cells. At a concentration of 100 µg/mL, it displayed only minor toxicity and reduced the number of viable cells in the CCD 841 CoTr line to 74% (NR) and 89% (MTT) compared to the untreated control. In turn, at this concentration, the HT29 cells decreased to 57% in the NR and 68% in the MTT assays. At 200 µg/mL, similar toxicity was observed against both cell lines and the cell viability decreased below 40%. The HCV fraction also exhibited cytotoxicity, but only in the case of normal cells; therefore, it was not included in further investigations.

### 3.2. Subfractions

Based on the MTT and NR results, the most active EaCV fraction was subjected to further fractionation using 20% methanol (EaCV_1), 60% methanol (EaCV_2), and 100% methanol (EaCV_3). The subfractions were studied in terms of their biological activity and phytochemical composition.

#### 3.2.1. Biological Assay

**Figure 2:**
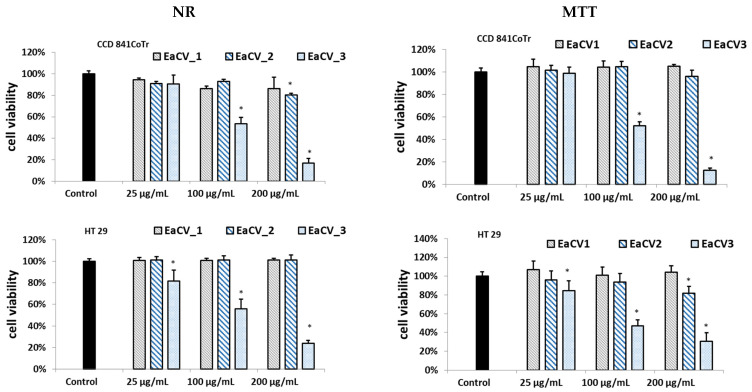
Effect of the different concentrations of subfractions obtained from ethyl acetate extract of *Carlina vulgaris* (EaCV) on cell viability determined by the NR and MTT assays, expressed as a % of the control (0.5% of DMSO in medium). The fractions were obtained using 20% methanol (EaCV_1), followed by 60% methanol (EaCV_2) and 100% methanol (EaCV_3). The data are the mean (*n* = 3) ± SD. One-way ANOVA followed by Dunnett’s post hoc test; the differences between the samples and the control were considered significant at *p* < 0.05. * Indicates statistically significant difference.

The results from the MTT and NR assays are shown in Figure 2.

As can be seen, EaCV_3 exhibited significant cytotoxicity on both cell lines. However, for HT29, cytotoxicity was observed across all the tested concentration ranges. Conversely, the subfraction at 25 µg/mL did not have a negative effect on the CCD 841 CoTr. Cell viability remained above 94%, whereas in HT29, the number of viable cells decreased to approximately 80%.

**Figure 3:**
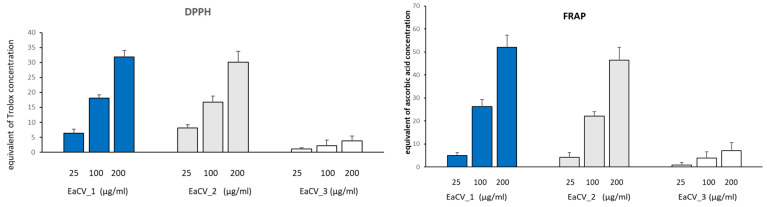
The results (±SD) on the radical scavenging activity (DPPH) and ferric reducing antioxidant power (FRAP) for subfractions obtained from ethyl acetate (EaCV) fractions of the extract from *C. vulgaris*. Equivalent of ascorbic acid/Trolox, the reducing/antioxidant power of the extract at a given concentration is equivalent to the reducing power of a given concentration of ascorbic acid/Trolox equivalent of ascorbic acid concentration.

The free radical scavenging capacity (based on the DPPH) and the ferric reducing antioxidant power (FRAP) of the tested samples were also assessed (Figure 3)

EaCV_1 and EaCV_2 exhibited similar free radical scavenging activity and ferric reducing ability. At a concentration of 100 µg/mL, their antioxidant potential was equivalent to 22–26 µg/mL ascorbic acid and 17–18 µg/mL Trolox. This suggests the presence of compounds with high antioxidant potential in these subfractions. On the other hand, EaCV_3 showed only a minor antioxidant effect.

#### 3.2.2. Phytochemical Characterization of the Subfractions

The subfractions were characterized based on UHPLC–DAD–MS analysis (Figures S2 and S3). The identified components were quantified, and the results are summarized in Table 1.

The results from the chromatographic analysis revealed that the first fraction (EaCV_1) primarily contained phenolic acids, with predominant chlorogenic acids, followed by densifloside. In turn, the second fraction exhibited a high concentration of flavonoids, such as C-glycosides of apigenin, followed by C-glycosides of luteolin, rutin, and nicotiflorin. Within the methanolic fraction (EaCV_3), only oxylipins were identified, constituting 40% of the fraction. As anticipated, EaCV_1 and EaCV_2, which had the highest polyphenol content, demonstrated the most significant free radical scavenging activity in the DPPH test, along with the highest ferrous ion-reducing capability.

### 3.3. Isolation of the Target Compounds and Structure Elucidation

**Figure 4:**
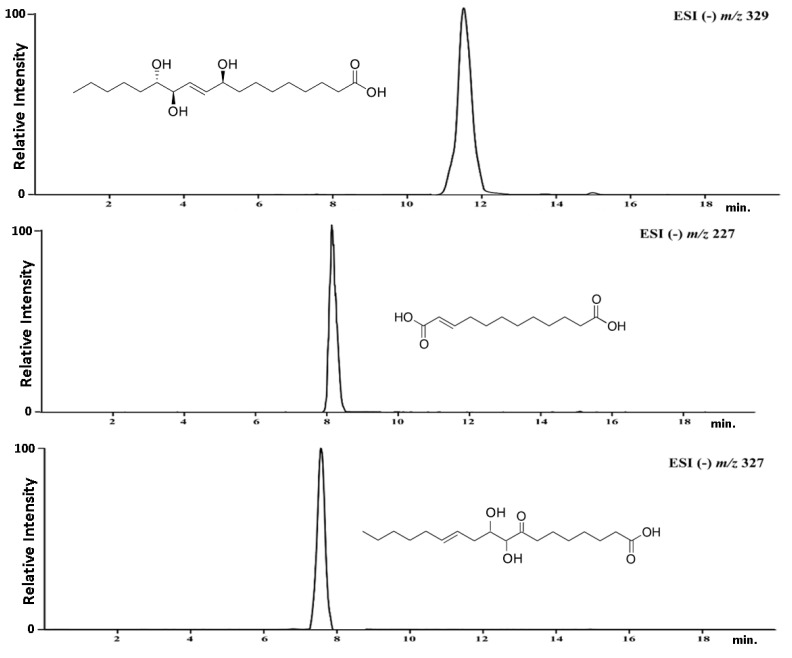
Chromatograms and structure of the isolated compounds: pinellic acid, traumatic acid, and 9,10-dihydroxy-8-oxsooctadec-12-enic acid.

The isolation of the compounds was carried out using a semi-preparative HPLC system, with optimized chromatographic conditions. This yielded three compounds, namely pinellic acid, traumatic acid, and 9,10-dihydroxy-8-oxsooctadec-12-enic acid (Figure 4).

The chemical structure of the compounds isolated from the EaCV_3 fraction was confirmed using high-resolution MS/MS (Figure S4) and NMR spectroscopy. Based on the obtained results, the structure of the isolated compounds were verified. The obtained results are presented below.

**Traumatic acid:**
  HR MS/MS: C_12_H_20_O_4_; *m*/*z* –H 227.1285 (M-H); 183.7652; 165.5362
  ^1^H NMR (500 MHz, MeOH–4D): δ (ppm) 7.11 (1H, dd, *J* = 8.1; 1.8 Hz H–3); 6.00 (1H, d, *J* = 8.1 Hz H–2), 2.30 (2H, t, *J* = 7.2 Hz H–11); 2.18 (2H, dd, *J* = 7.1; 1.8 Hz H–4); 1.52 (2H, dd, *J* = 7.1 Hz H–10); 1.29 (10H; s H–5; H–6; H–7; H–8; H–9)
  ^13^C NMR (125 MHz, MeOH–4D): δ (ppm) 178.4 (C–12); 171.5 (C–1); 153.6 (C–3); 120.4 (C–2); 34.0 (C–11); 33.1 (C–4); 29.6 (C–5; C–6; C–7; C–8; C–9); 24.7 (C–10).
**Pinellic acid:**
  HR MS/MS: C_18_H_34_O_5_; *m*/*z*-H 329.2337 (M-H); 229.3276; 211.3278; 171.7682
  ^1^H NMR (500 MHz, MeOH–4D): δ (ppm) 5.72 (1H; dd; *J* = 15.6; 5.1; Hz; H–10); 5.65 (1H; *J* = 15.6; 5.2 Hz; H–11); 4.05 (1H; m; H–12); 3.91 (1H; dd *J* = 5.5; 5.0 Hz; H–9); 3.41 (1H; m; H–13); 2.27 (2H; t; *J* = 7.6 Hz; H–2); 1.52 (2H; m; H–3); 1.48 (2H; m; H–8); 1.44 (2H, m, H–14); 1.31 (2H; m; H–17); 1.29 (4H; m; H–4; H–5); 1.25 (8H; m; H–6; 7; 15; 16), 0.89 (3H; t, *J* = 6.3 Hz; H–18).
  ^13^C NMR (125 MHz, MeOH–4D): δ (ppm) 177.6 (C–1); 136.6 (C–10); 131.2 (C–11); 76.4 (C–13); 75.8 (C–12); 73.0 (C–9); 38.3 (C–8); 35.1 (C–2); 33.6 (C–14); 33.2 (C–16); 30.5 (C–6); 30.4 (C–5); 30.1 (C–4); 26.6 (C–15); 26.5 (C–7); 26.1 (C–3); 23.7 (C–17); 13.9 (C–18).
**9,10-dihydroxy-8-oxsooctadec-12-enic acid:**
  HR MS/MS: C_18_H_32_O_5_; *m*/*z*-H 327.2180; 211.3278; 171.7682
  ^1^H NMR (500 MHz, MeOH–4D): δ (ppm) 5.72 (1H; dd; *J* = 15.6; 5.1; Hz; H–12); 5.65 (1H; *J* = 15.6; 5.2; H–13); 4.05 (1H; m; H–10); 3.91 (1H; dd *J* = 5.5; 5.0 Hz; H–9); 2.45 (2H; t; *J* = 7.1 Hz; H–7); 2.30 (2H; m; H–2); 2.23 (1H; m; H–11); 2.18 (1H; m; H–14), 1.98 (1H; m; H–11); 1.57 (4H, m; H–3; 6), 1.31 (2H; m; H–17); 1.29 (8H; m; H–4; 5; 15; 16); 0.90 (3H; t; *J* = 6.3 Hz; H–18)
  ^13^C NMR (125 MHz, MeOH–4D): δ (ppm) 204.2 (C–8);178.4 (C–1); 133.6 (C–13); 124.2 (C–12); 83.4 (C–9); 64.7 (C–10); 43.8 (C–7); 34.3 (C–2); 33.7 (C–14); 31.9 (C–16); 30.4 (C–11); 29.6 (C–15); 29.0 (C–6); 28.8 (C–5); 27.6 (C–4); 24.7 (C–3); 22.8 (C–17); 14.1 (C–18).

### 3.4. Biological Assay of the Isolated Compounds

**Figure 5:**
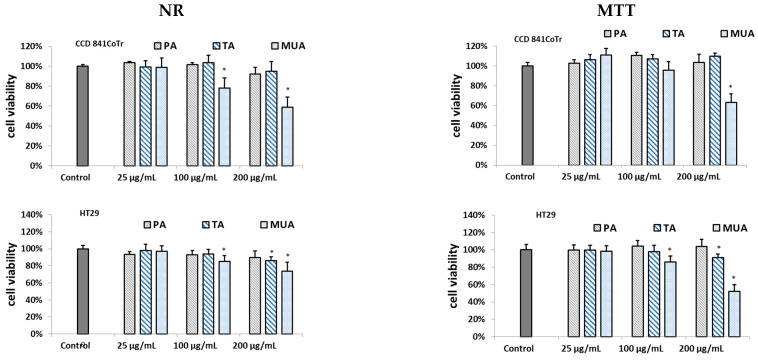
Effect of the different concentrations of pinellic acid (PA), traumatic acid (TA), and 9,10-dihydroxy-8-oxsooctadec-12-enic acid (MUA) on cell viability determined by the NR and MTT assays, expressed as a % of the control (0.5% of DMSO in medium). The data are the mean (*n* = 3) ± SD. One-way ANOVA followed by Dunnett’s post hoc test; the differences between the samples and the control were considered significant at *p* < 0.05. * Indicates statistically significant difference.

The results on the cytotoxic effect of the isolated compounds are shown in Figure 5. Among the isolated compounds, only 9,10-dihydroxy-8-oxsooctadec-12-enic acid (MUA), belonging to mono-unsaturated fatty acids, exhibited significant cytotoxicity, though without selectivity. At a dose of 200 µg/mL, it reduced the number of viable cells for both the cancer and reference cell lines to approximately 50% compared to the control. On the other hand, traumatic acid (TA) displayed weak cytotoxicity on the HT29 line, but only at a dose of 200 µg/mL, with viability dropping to about 85% compared to the control. Pinellic acid (PA) did not show activity (Figure 5).

**Figure 6:**
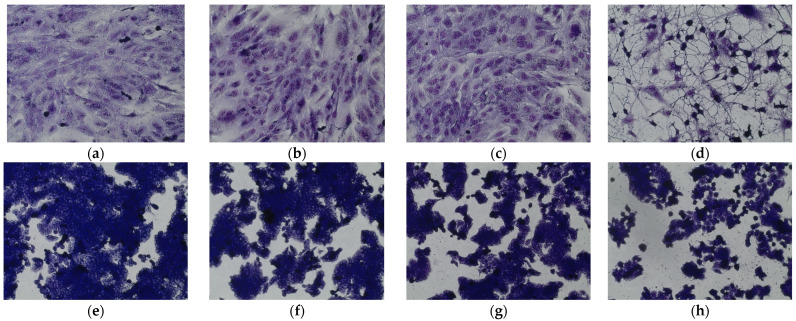
May–Grünwald–Giemsa (MGG) staining of (**a**–**d**) the normal human colon epithelial cell line (CCD 841 CoTr) and (**e**–**h**) human colorectal adenocarcinoma (HT29). Incubated with pinellic acid (**b**,**f**), traumatic acid (**c**,**g**), and 9,10-dihydroxy-8-oxsooctadec-12-enic acid (**d**,**f**) at a concentration of 200 µg/mL, control (**a**,**e**). Magnification 200×. Bar = 20 µm.

The cell culture analysis, after incubation with the tested substances, showed changes in the cell morphology, depending on the compound used (Figure 6). It was found that pinellic acid and traumatic acid did not significantly affect the morphology of both types of cells. On the other hand, 10-dihydroxy-8-oxsooctadec-12-enic acid, affected the growth of the culture by detaching cells from the adhesive surface. In addition, the rounding of cells and their separation from each other can be observed (HT29). Normal intestinal epithelial cells (CCD 841 CoTr) were also sensitive to the activity of the tested compound. At the highest concentration (200 µg/mL), the cells shrank, leaving only the cytoplasmic lining connecting neighboring cells. In addition, as it was observed in the case of tumor cell culture, a reduced number of cells was found compared to the control.

**Figure 7:**
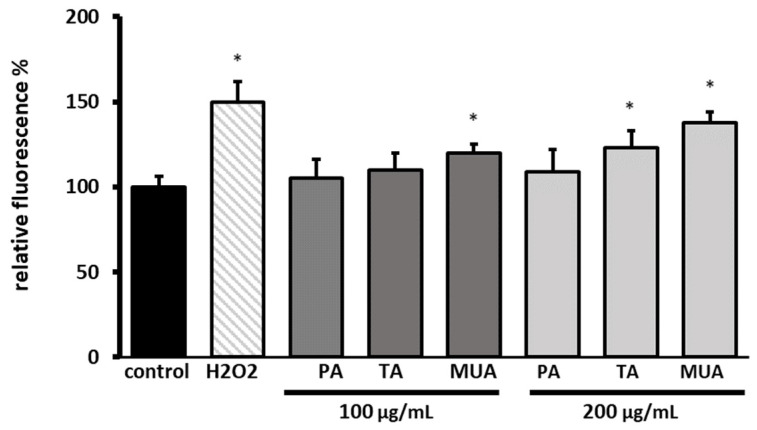
Relative fluorescence of 2′,7′-dichlorodihydrofluorescein (DCF) in human colorectal adenocarcinoma (HT29), calculated as a percentage, in comparison with the untreated control cells. * Indicates a statistically significant difference (*p* < 0.05) versus the untreated controls. H_2_O_2_ was used as a positive control. The data are the mean ± SD (*n* = 3). One-way ANOVA followed by Dunnett’s multiple comparison post hoc test.

The antioxidant capacity studies indicated that the compounds do not possess free radical scavenging ability or the ability to reduce iron ions (Table S1). In fact, at a concentration of 200, both TA and MUA even increased intracellular ROS production (Figure 7).

## 4. Discussion

Plants can be a source of new therapeutic agents and, therefore, research on the activity of plants can help discover new molecules with biological properties beneficial for human health [19]. *Carlina vulgaris* is a plant with unexplored potential and, still, little is known about its activity, despite the mention on its utility in ethnomedicine [15].

In our study, we investigated the cytotoxicity of fractions obtained from a *C. vulgaris* extract against human colorectal adenocarcinoma cells (HT29), using a normal human colon epithelial cell line (CCD 841 CoTr) as a reference. A bioactivity-guided approach led to the isolation of three main constituents from the extract with potential cytotoxic effects on cells, namely pinellic acid (PA), traumatic acid (TA), and 9,10-dihydroxy-8-oxsooctadec-12-enic acid (MUA). The compounds belong to oxylipin family, which are products from the oxygenation of fatty acids accumulated in plants in response to stress conditions [20,21]. They exhibit interesting biological activities, including pro-/anti-inflammatory, antimicrobial, neuroprotective, and regulatory effects [22,23]. However, their actions depend on the chemical structure, and some opposite effects have been found for omega-3 and omega-6 oxylipins. For example, the former are anti-inflammatory and inhibit tumor development, whereas the latter induce inflammation and promote cancer growth [24]. The effect of oxygenated fatty acids on cancer development has been the subject of numerous studies [25,26,27]. However, little is still known about the action of specific compounds from this group.

The current study revealed that pinellic acid did not demonstrate activity against HT-29, consistent with the existing literature indicating either its lack of activity or only mild cytotoxicity. Both PA and the other structurally similar omega-6 fatty acid showed no cytotoxicity against other types of cancer cell lines, such as lung adenocarcinoma, skin melanoma, adenosquamous carcinoma, and human glioblastoma [23,28]. Only moderate cytotoxic activity by PA was noted against hepatic cell carcinoma and prostate cancer [29]. On the other hand, traumatic acid at a concentration of 200 µg/mL was not cytotoxic against normal cells and displayed a mild cytotoxic effect on HT29. Jabłońska-Trypuć et al. found that TA was also effective against human breast cancer [30]. Among the isolated compounds, 9,10-dihydroxy-8-oxsooctadec-12-enic acid exhibited the highest cytotoxic activity; however, it was not selective and decreased viability in both normal and cancer cells. Although there are no literature data on the impact of this acid on cells, it has been observed that certain long chain fatty acids (LCFAs) demonstrate pro-apoptotic activities linked to lipid peroxidation, disruptions in the cell cycle, and modifications in gene expression associated with cell apoptosis. In addition, LCFAs are involved in the disturbance of various signaling pathways. For example, they may suppress phosphoinositide 3-kinases, which participate in the growth, proliferation, and differentiation of cancer cells. As a result, they might serve as adjunctive chemotherapeutics in cases of colorectal cancer [31,32].

The cytotoxicity of the isolated compounds was likely a result of their impact on intracellular ROS production. After treating the cells with TA and MUA, the ROS levels increased, inducing oxidative stress and disrupting the redox systems in the organism. Such an effect is commonly regarded as one of the mechanisms of anticancer activity for many substances [33,34]. In a previous study by Jabłońska-Trypuć et al., it was evidenced that TA stimulates ROS generation in cells, disturbs the ratio of GSH/GSSH (reduced and oxidized glutathione), increases caspase 7 activity, and induces membrane lipid peroxidation [30].

It should also be noted that a low concentration of oxylipins, corresponding to the content in the extract, were not active. This suggests that the cytotoxicity of *C. vulgaris* may result from the presence of unknown components. Since they have no UV–Vis absorption and are not ionizable, they may not be detected using DAD and MS. Therefore, it is worthwhile to continue investigating this plant.

A limitation of this study is the relatively short incubation time of the cells with the isolated compounds. In order to fully assess the cytotoxicity of the oxylipins against human colorectal adenocarcinoma, cell viability should also be evaluated during longer incubation periods, including 48 and 72 h.

## 5. Conclusions

This study evaluated the cytotoxic effects of *C. vulgaris* on human colorectal adenocarcinoma (HT29) cells. Employing a bioactivity-guided approach, the research led to the isolation of oxylipins, such as traumatic acid, pinellic acid, and 9,10-dihydroxy-8-oxo-octadec-12-enic acid, with potential cytotoxic activities. Among them, the last compound displayed significant cytotoxicity, whereas traumatic acid showed mild cytotoxicity. Both compounds lacked selectivity and decreased the viability of both cancer and normal cells. These cytotoxic effects were found to be associated with the generation of intracellular reactive oxygen species. Further investigation is required to elucidate the mechanisms of action of these components. Additionally, exploring the cytotoxicity on other cancer cell lines would be worthwhile.

## Figures and Tables

**Table 1:** Quantitative analysis of the subfractions obtained using 20% methanol (EaCV_1), followed by 60% methanol (EaCV_2), and 100% methanol (EaCV_3); expressed as mg per gram of the dried extract (±SD).

No.	Compound	Fraction Amount (mg/g d.m. of Fraction)		
		EaCV_1	EaCV_2	EaCV_3
1	3-caffeoquinic acid	23.20 ± 0.13	ND	ND
2	5-caffeoquinic acid	157.69 ± 0.13	ND	ND
3	densifloside	113.73 ± 0.28	ND	ND
4	carlinoside	ND	167.27 ± 0.66	ND
5	schaftoside	ND	84.25 ± 0.07	ND
6	isoschaftoside I	ND	207.09 ± 0.05	ND
7	vitexin	ND	214.98 ± 0.39	ND
8	apigenin di-C arabinoside	ND	2.68 ± 0.05	ND
9	taxifolin	ND	3.38 ± 0.30	ND
10	rutin	ND	69.20 ± 0.32	ND
11	nicotiflorin	ND	52.66 ± 0.31	ND
12	traumatic acid	ND	ND	137.04 ± 5.77
13	9,10-dihydroxy-8-oxsooctadec-12-enic acid	ND	ND	124.99 ± 0.07
14	pinellic acid	ND	ND	140.71 ± 0.42

## Data Availability

The data are contained within the article or the Supplementary Materials.

## Supplementary Materials

The following supporting information can be downloaded at: https://www.mdpi.com/article/10.3390/antiox12091704/s1, Scheme S1. The schematic diagram of the extraction procedure. Figure S1. Effect of the different concentrations of extracts obtained from *Carlina vulgaris* on cell viability determined by the MTT assay. Figure S2. The UHPLC-ESI-MS(-) chromatograms subfractions from the extract of *C. vulgaris*. Figure S3. The UHPLC-DAD chromatograms subfractions from the extract of *C. vulgaris* obtained at a wavelength of 254 nm. Figure S4. Exemplary MS spectra for target compounds (traumatic acid, 9,10-dihydroxy-8-oxsooctadec-12-enic acid, pinellic acid). Table S1. The results of radical scavenging activity (DPPH) and ferric reducing antioxidant power (FRAP) obtained for pinellic acid (PA), traumatic acid (TA), and 9,10-dihydroxy-8-oxsooctadec-12-enic acid (MUA). Values are the mean ± standard deviation (SD) in triplicate.
