# Supplementary material for: Antioxidant and Cytotoxic Potential of Carlina vulgaris Extract and Bioactivity-Guided Isolation of Cytotoxic Components
Source: Antioxidants (Basel). 2023 Sep 1;12(9):1704. doi: 10.3390/antiox12091704 (PMC10525758; doi:10.3390/antiox12091704)
Supplement: Supplementary file 1 [file antioxidants-12-01704-s001.zip › antioxidants-2579159-supplementary.pdf]

antioxidants

Supplementary material

Antioxidant and Cytotoxic Potential of *Carlina vulgaris* Extract and Bioactivity-Guided Isolation of Cytotoxic Components

Ireneusz Sowa [1,*], Roman Paduch [2], Jarosław Mołdoch [3], Dariusz Szczepanek [4], Jacek Szkutnik [5], Paweł Sowa [6], Katarzyna Tyszczuk-Rotko [7], Tomasz Blicharski [8] and Magdalena Wójciak[1,*]

1 Department of Analytical Chemistry, Medical University of Lublin, Chodźki 4a, 20-093 Lublin, Poland
2 Department of Virology and Immunology, Institute of Biological Sciences, Faculty of Biology and Biotechnology, Maria Curie-Skłodowska University, 19 Akademicka Street, 20-033 Lublin, Poland
3 Department of Biochemistry and Crop Quality, Institute of Soil Science and Plant Cultivation, State Research Institute, 24-100 Puławy, Poland
4 Chair and Department of Neurosurgery and Paediatric Neurosurgery, Medical University of Lublin, 20-090 Lublin, Poland
5 Independent Unit of Functional Masticatory Disorders, Medical University of Lublin, 20-093 Lublin, Poland
6 Department of Otorhinolaryngology and Oncological Laryngology, Faculty of Medical Sciences in Za-brze, Medical University of Silesia in Katowice, 40-055 Katowice, Poland
7 Institute of Chemical Sciences, Faculty of Chemistry, Maria Curie-Skłodowska University in Lublin, 20-031 Lublin, Poland
8 Department of Orthopaedics and Rehabilitation, Medical University of Lublin, Lublin, Poland

* Correspondence: ireneusz.sowa@umlub.pl (I.S.); magdalena.wojciak@umlub.pl (M.W.)

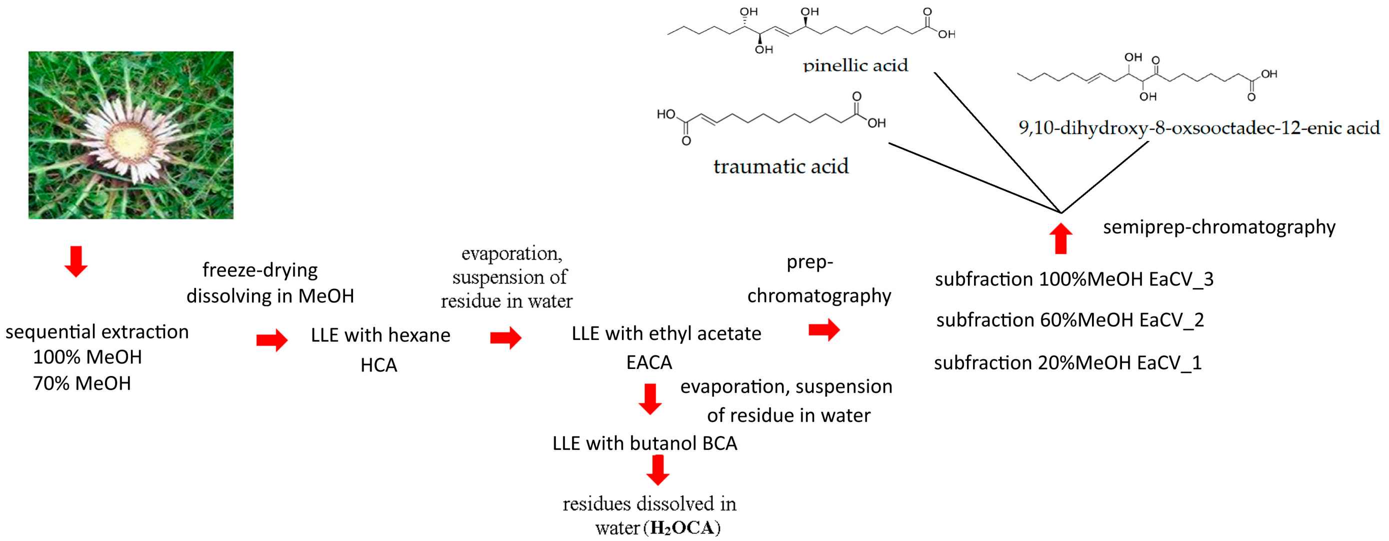

Scheme S1. The schematic diagram of the extraction procedure.

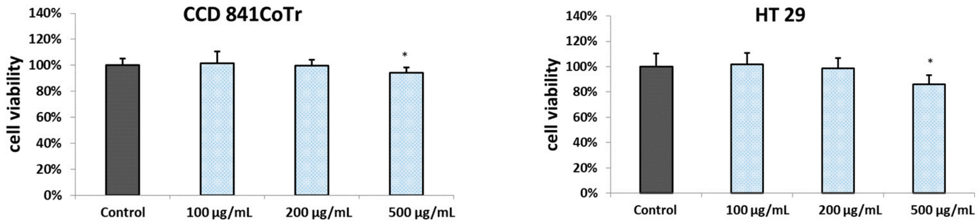

Figure S1. Effect of the different concentrations of extracts obtained from *Carlina vulgaris* on cell viability determined by the MTT assay and expressed as a % of control (0.5% of DMSO in medium). The data are means (n = 3) ± SD. One-way ANOVA followed by Dunnett¬s post hoc test; the differences between samples and control were considered significant at p < 0.05. * indicates statistically significant difference**.**

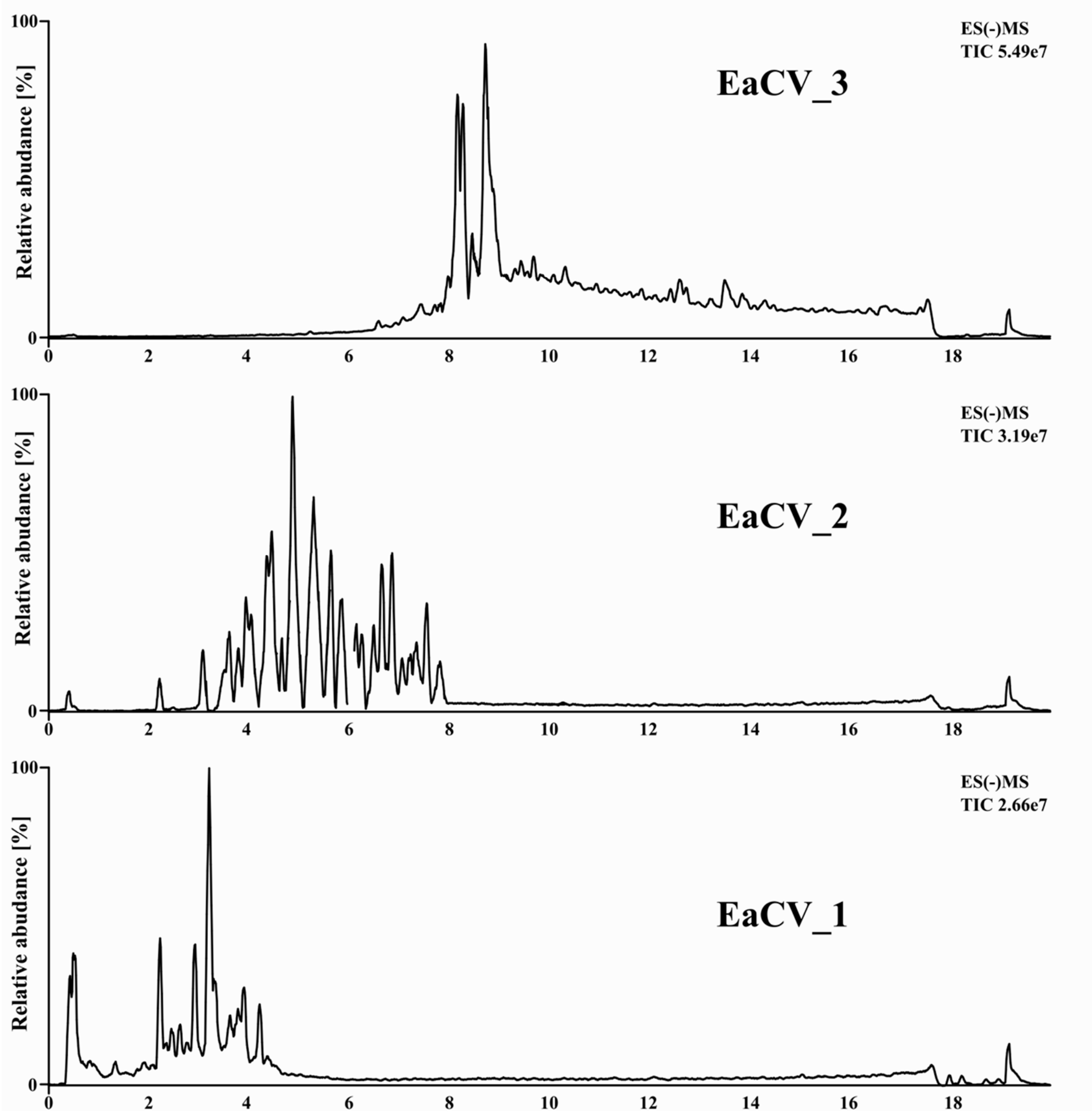

Figure S2. The UHPLC - ESI - MS(-) chromatograms sub-fractions from the extract of *C. vulgaris*.

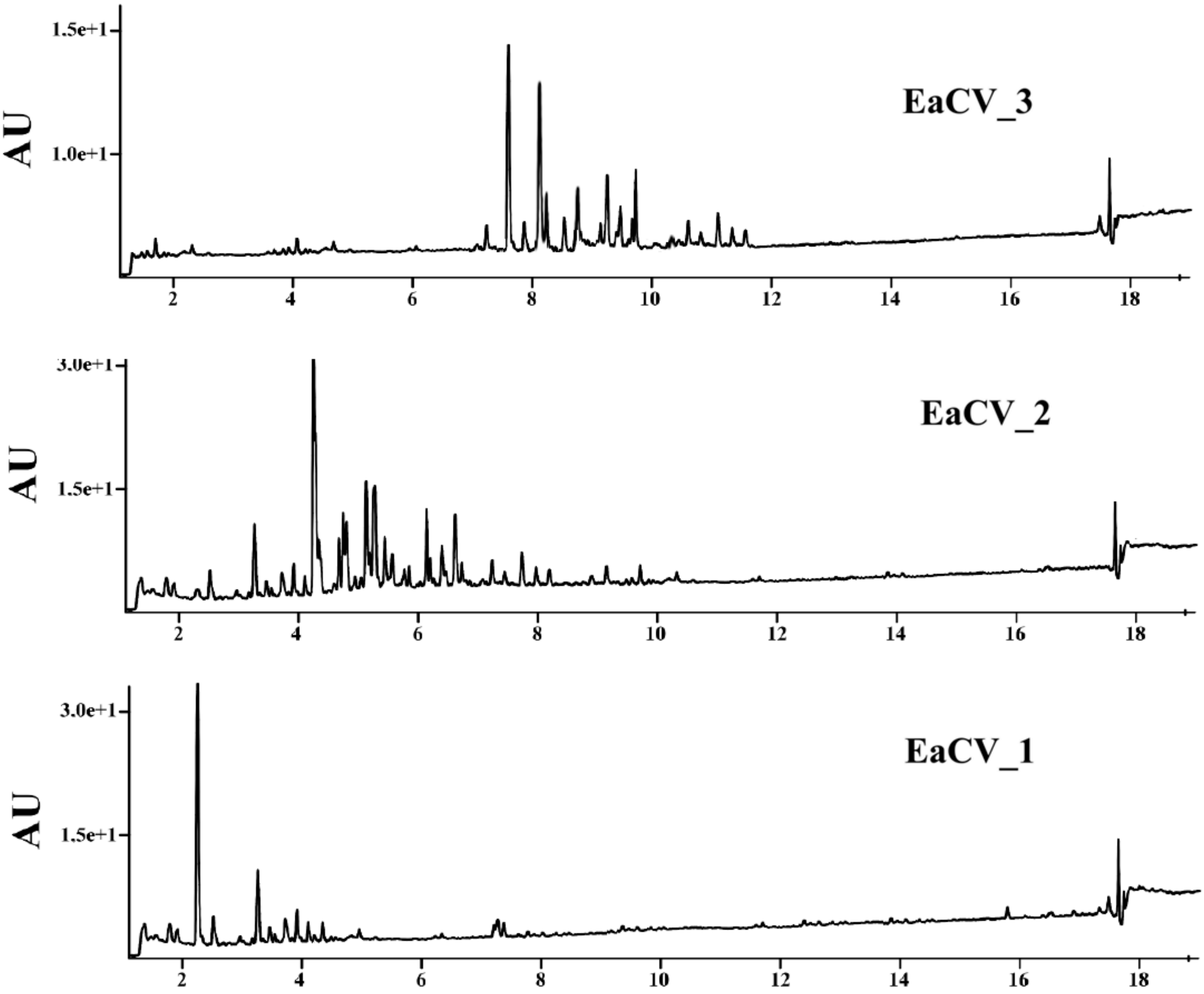

Figure S3. The UHPLC-DAD chromatograms sub-fractions from the extract of *C. vulgaris* obtained at wavelength of 254 nm.

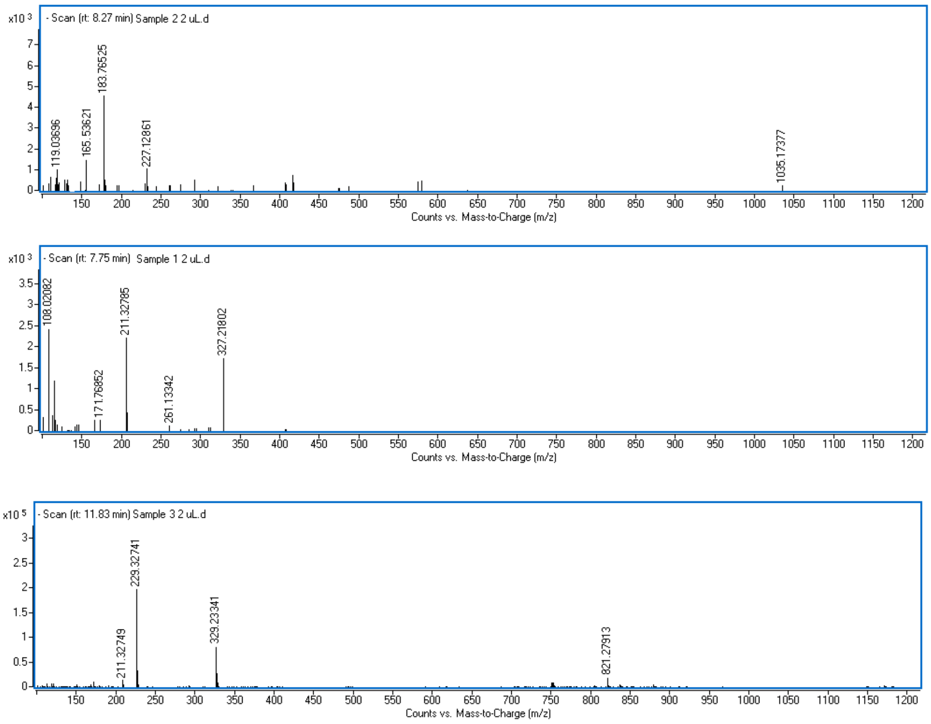

Figure S4. Exemplary MS spectra for target compounds (traumatic acid, 9,10-dihydroxy-8-oxsooctadec-12-enic acid, pinellic acid)**.**

Table S1. The results of radical scavenging activity (DPPH) and ferric reducing antioxidant power (FRAP) obtained for pinellic acid (PA), traumatic acid (TA) and 9,10-dihydroxy-8-oxsooctadec-12-enic acid (MUA). Values are means ± standard deviation (SD) of triplicate.

compound	concentration (µg/ml)	Equivalent of Trolox concentration (DPPH)	Equivalent of ascorbic acid concentration (DPPH)
	25	2.169 ± 0.204	0.405 ± 0.144
PA	100	2.588 ± 0.301	1.351 ± 0.144
	200	3.309 ± 0.845	2.500 ± 0.125
	25	0.797 ± 0.516	0.372 ± 0.096
TA	100	1.762 ± 0.381	1.554 ± 0.125
	200	2.030 ± 0.344	3.014 ± 0.125
	25	0.623 ± 0.288	0.912 ± 0.304
MUA	100	2.693 ± 0.418	3.581 ± 0.280
	200	4.030 ± 0.358	6.791 ± 0.201
